# Associations between body composition profile and hypertension in different fatty liver phenotypes

**DOI:** 10.3389/fendo.2023.1247110

**Published:** 2023-11-28

**Authors:** Xiaoyin Huang, Yuchen Zeng, Mingyang Ma, Liangguang Xiang, Qingdan Liu, Ling Xiao, Ruimei Feng, Wanxin Li, Xiaoling Zhang, Moufeng Lin, Zhijian Hu, Hongwei Zhao, Shanshan Du, Weimin Ye

**Affiliations:** ^1^ Department of Epidemiology and Health Statistics, School of Public Health, Fujian Medical University, Fuzhou, China; ^2^ Department of Ultrasonography, The Affiliated Fuqing Hospital of Fujian Medical University, Fuqing, China; ^3^ Department of General Surgery, The Affiliated Fuqing Hospital of Fujian Medical University, Fuqing, China; ^4^ Department of Epidemiology, School of Public Health, Shanxi Medical University, Taiyuan, China; ^5^ Department of Public Health, The Fifth Hospital of Fuqing City, Fuqing, China; ^6^ Department of Epidemiology and Biostatistics, School of Public Health, Texas A&M University, College Station, TX, United States; ^7^ Department of Medical Epidemiology and Biostatistics, Karolinska Institutet, Stockholm, Sweden

**Keywords:** hypertension, body composition, fatty liver disease, phenotype, obesity, lipid, mediation analysis, association

## Abstract

**Background:**

It is currently unclear whether and how the association between body composition and hypertension varies based on the presence and severity of fatty liver disease (FLD).

**Methods:**

FLD was diagnosed using ultrasonography among 6,358 participants. The association between body composition and hypertension was analyzed separately in the whole population, as well as in subgroups of non-FLD, mild FLD, and moderate/severe FLD populations, respectively. The mediation effect of FLD in their association was explored.

**Results:**

Fat-related anthropometric measurements and lipid metabolism indicators were positively associated with hypertension in both the whole population and the non-FLD subgroup. The strength of this association was slightly reduced in the mild FLD subgroup. Notably, only waist-to-hip ratio and waist-to-height ratio showed significant associations with hypertension in the moderate/severe FLD subgroup. Furthermore, FLD accounted for 17.26% to 38.90% of the association between multiple body composition indicators and the risk of hypertension.

**Conclusions:**

The association between body composition and hypertension becomes gradually weaker as FLD becomes more severe. FLD plays a significant mediating role in their association.

## Introduction

Body mass index (BMI), Quetelet’s normalization of body weight (kg) by height squared (m^2^), a traditional diagnosis and understanding of the pathophysiology of obesity, is still widely applied today in quantitative studies on the effects of body mass on health (1, 2). However, with the prevailing notions of obese phenotypes, such as normal-weight obese, metabolically obese with normal weight, metabolically healthy obese, and metabolically unhealthy obese, BMI shows apparent limitations in our comprehensive understanding of obesity-associated metabolic disturbances (3–5). Body composition, the quantitative and qualitative analysis of lean and adipose tissue compartments, has been suggested to provide insight into both nutritional status and functional capacity of the whole body (1, 6, 7).

With the increasing body weight and aging worldwide, a great variation can be observed in body composition. More and more attention has been paid to its association with multiple metabolic disorders (8). A more precise assessment of body mass is essential for the more effective management of the obesity epidemic. Currently, BMI and body fat have been evidenced to be independent risk factors for hypertension, and a few studies have also reported positive associations between body fat, central obesity (9, 10), skeletal muscle, and hypertension (11, 12). However, the systemic description of body composition on hypertension risk is limited, and the characteristic body composition indicators in hypertension risk are still ambiguous, especially in Asia, where the population has a lower BMI (13) but shows a comparable or higher risk of multiple metabolic diseases compared with European and American populations (14, 15).

In addition, obesity is also a confirmed risk factor of fatty liver disease (FLD) (16), and FLD has been reported to be an independent risk factor and an important driving force in the development and progression of hypertension (17). On the other hand, previous studies have reported different risks of cardiovascular diseases in normal-weight and overweight/obese non-FLD and FLD patients (18–20). Yet, it needs further exploration to clarify whether FLD affects the association between body composition characteristics and hypertension.

Therefore, we conducted a cross-sectional study among the general population in southeast China and aimed to explore the characteristic body composition profile of hypertensive patients and further investigate whether and how the association between body composition and hypertension varied with the presence and severity of FLD, trying to provide clues on the clinical application of body composition.

## Materials and methods

### Study design and population

The Fuqing Cohort Study is an ongoing, prospective population-based cohort study in Fuqing City, Fujian Province, located in a coastal area of southeast China. Local residents aged 35–75 years were recruited. The first wave of the cohort baseline enrolment was conducted in 2019. The second wave with more comprehensive data collection was initiated in July 2020 and will continue until 50,000 residents are recruited. In the current study, we included all participants (*n* = 7,662) from Gaoshan Town of Fuqing City from July 2020 to June 2021 in the baseline survey of the Fuqing Cohort Study. The study has been approved by the Ethics Review Committee of Fujian Medical University (approval numbers [2017-07] and [2020-58]), and the study protocol conforms to the ethical guidelines of the 1975 Declaration of Helsinki. Written informed consent was obtained from all participants.

**Figure 1:**
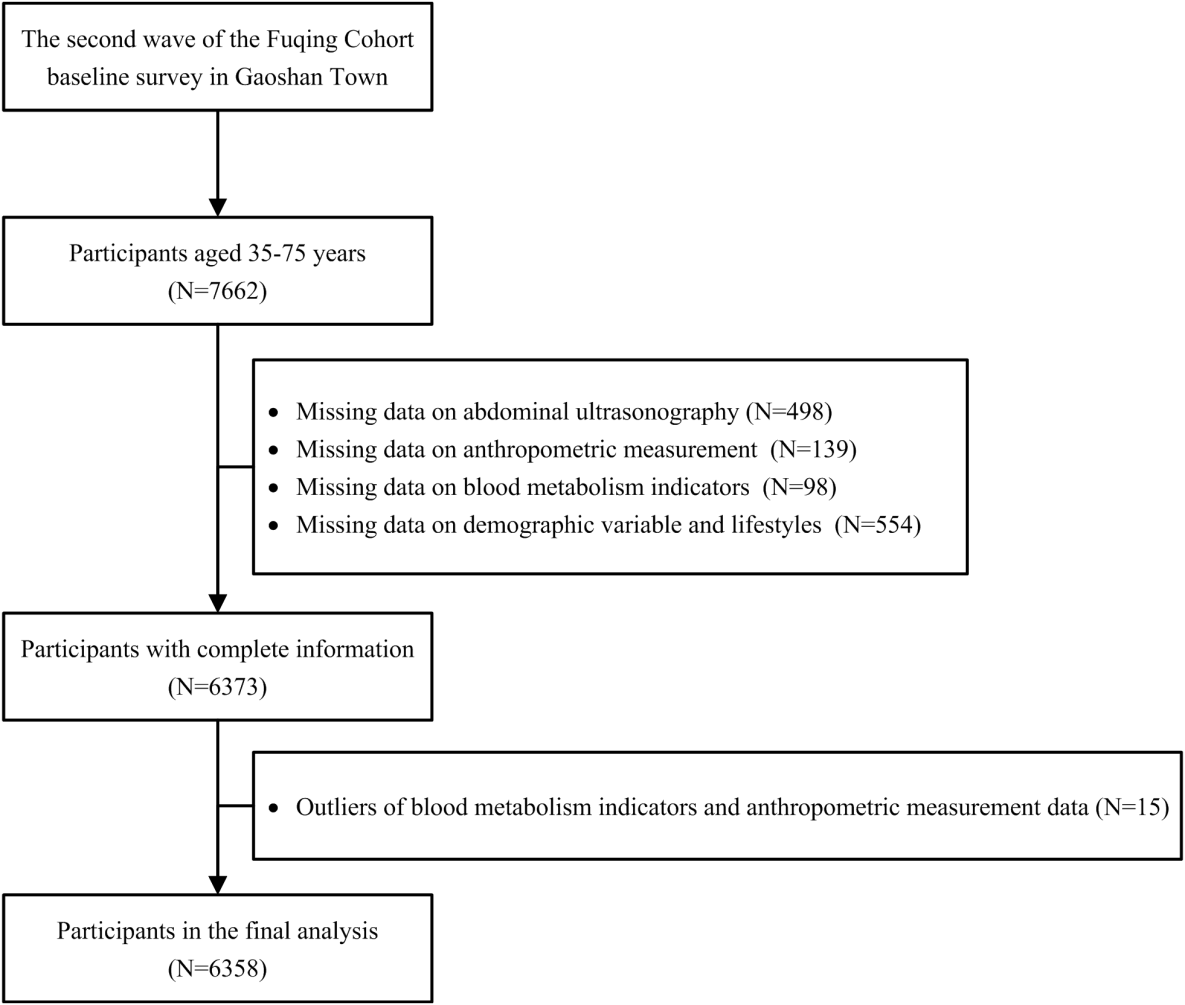
Flowchart of the selection of study participants.

Abdominal ultrasonography was conducted among all participants since the second wave. After excluding participants with missing data on abdominal ultrasonography, anthropometric measurement, blood metabolism indicators, demographic information, lifestyle variables, and outliers, we included 6,358 participants in the second wave of the Fuqing cohort eventually (**Figure 1**).

### Data collection

Each participant was invited to finish a face-to-face interview by trained and qualified interviewers using a computerized, structured questionnaire (https://cohort.fjmu.edu.cn/), and data on demographic and social–economical characteristics, history of disease and medication, and lifestyle information (tobacco use, alcohol drinking, and physical activity) were collected. Tobacco use was defined as smoking at least one cigarette per day for at least 6 months, and alcohol drinking was defined as at least once per week in the past year. The International Physical Activity Questionnaire-short form (IPAQ-SF) was applied, and physical metabolic equivalent (MET/day) was calculated according to the IPAQ scoring protocol to estimate total physical activity (21).

Venous blood samples were obtained from all participants after at least 8h of fasting, and serum was separated and used to determine the levels of fasting blood glucose (FBG), triglyceride (TG), total cholesterol (TC), high-density lipoprotein cholesterol (HDL-c), and low-density lipoprotein cholesterol (LDL-c), using standard laboratory procedures (Toshiba automatic biochemical analyzer, TBA-120FR, Japan). Fasting insulin level (FINS) was measured by electrochemiluminescence immunoassay (Roche Diagnostics, Cobas e 602, Germany).

### Anthropometric measurements

All measurements were conducted by trained staff according to standard protocol, and all participants were asked to wear light clothing and stand upright barefoot. Height, waist, and hip circumferences (WC and HC) were measured using a standard stadiometer and a tape meter (0.1 cm precision). WC was taken at the midway between the lowest rib and the top of the iliac crest, and HC was taken at the largest circumference of the buttocks. Body weight and composition metrics, including body fat percentage (BFR), body moisture rate (BMR), skeletal muscle (SM), and bone weight, were measured by a digital scale (0.1 kg precision, Tanita bioimpedance analyzer, BC-601, Japan), which utilizes bioelectrical impedance technology. BMI was defined as the body weight in kilograms divided by the square of the body height in meters. The waist-to-hip ratio (WHR) was calculated by dividing the WC by HC, and the waist-to-height ratio (WHtR) was determined by dividing the WC by height. Fat tissue index (FTI) (22), visceral adiposity index (VAI) (23), lipid accumulation product (LAP) (24), cardiometabolic index (CMI) (25), and lean tissue index (LTI) (22) were calculated according to reported equations.

Systolic blood pressure (SBP) and diastolic blood pressure (DBP) were measured with an electronic sphygmomanometer (Omron Company, OMRON U30, Kyoto, Japan) on the right arm in a semi-flexed position at the heart level after 5 min of seated rest. Two measurements were recorded, and the third is recorded if the difference between the two measurements is higher than 5 mmHg. The average of the two closet readings was calculated for analysis.

### Disease definitions

According to BMI criteria proposed for the Chinese population, under and normal weight (<24.0 kg/m^2^), overweight (24.0 to <28.0 kg/m^2^), and obesity (≥28.0 kg/m^2^) were defined.

Hypertension was defined as SBP ≥140 mmHg, DBP ≥90 mmHg, self-reported hypertension, or under antihypertensive treatment.

Ultrasonography was performed on all participants by experienced sonographers who were unaware of the clinical or laboratory data of the participants using ultrasound scanners (Hitachi Aloka Medical, ProSounda α7, Japan). FLD was diagnosed according to the standard criteria issued by the Fatty Liver Disease Study Group of the Chinese Liver Disease Association (26, 27).

### Statistical analysis

Continuous variables are shown as mean ± standard deviation (SD). Categorical variables are shown as percentages, and the chi-squared test was used to compare the differences between groups. Logistic regression models were constructed in several steps, and odds ratios (ORs) and 95% confidence intervals (CIs) were estimated. First, a crude model was built between body composition indicators, FLD, and hypertension, respectively. Then, age and sex were adjusted in the model. Third, BMI, current alcohol drinking, current smoking, and physical activity were further adjusted.

Stratified analyses were conducted to explore potential age- and sex-related interaction effects. Stratified analyses were also applied to explore the potential effects of body composition indicators on hypertension risk by the presence and severity of FLD. First, all participants were grouped into non-FLD, mild FLD, and moderate/severe FLD groups, and multivariable-adjusted logistic regression models were constructed between body composition and hypertension. To fit trends between each indicator and the corrected risk of hypertension in each group, the multivariable-adjusted logistic regression models were constructed between body composition and hypertension with interactions. The predicted hypertension risks versus each body composition indicator were calculated after all confounding factors were fixed at their reference levels.

Then, all participants were regrouped according to the recommended cutoff or tertiles of body composition indicators to explore the association between FLD and hypertension in the context of levels of body composition indicators. Logistic regression models were constructed between FLD and hypertension.

We examined potential multiplicative interactions between FLD and body composition indicators. The presence of multiplicative interaction was explored by introducing a cross-product term in the regression model and the *P*-value was derived by the Wald test.

To explore the potential mediating effect of FLD on the relationship between each body composition indicator and hypertension, we performed a mediation analysis using the counterfactual framework method. The PARAMED module in Stata was used to estimate the total associations and natural direct and indirect associations. The proportion mediated was calculated log(natural indirect effect) /log(total effect). To fit the module, the FLD level was further classified as having FLD or not.

Previous studies have suggested that FLD is associated with cholecystectomy, and patients who underwent cholecystectomy were more than twice as likely to have fatty liver disease than those who had not undergone cholecystectomy (28). Therefore, we explored the association between cholecystectomy and FLD as well as hypertension and performed a sensitivity analysis by excluding participants who underwent cholecystectomy.

All analyses were performed with SAS 9.4 statistical software (SAS Institute Inc., Cary, NC, USA) and Stata/SE, version 16.0 statistical software (only for mediation analysis, StataCorp, TX, USA), and a two-tailed *P <*0.05 was considered statistically significant.

## Results

### Clinicodemographic characteristics of participants

A total of 6,358 individuals were enrolled in this analysis. The prevalence of hypertension was 46.7%, and the prevalence of mild FLD and moderate/severe FLD was 25.2% and 10.2%, respectively.

The clinicodemographic characteristics of the participants are presented in **Table 1**. The prevalence of hypertension in men was 50.8%, significantly higher than 44.4% in women. Compared with the normotensive, the characteristics of the hypertensive population included older age; higher BMI; lower education level; more alcohol drinker; higher central obesity and FLD severity; higher TC, TG, and LDL-c; and lower HDL-c.

**Table 1:** The clinicodemographic characteristics of the population based on the presence of hypertension.

	Total	Hypertension	
		No (*n* = 3,390)	Yes (*n* = 2,968)
Sex			
Male	2,245 (35.3)	1,104 (49.2)	1,141 (50.8)^**^
Female	4,113 (64.7)	2,286 (55.6)	1,827 (44.4)
Age, years			
<40	347 (5.5)	287 (82.7)	60 (17.3)^**^
40–49	1,124 (17.7)	840 (74.7)	284 (25.3)
50–59	2,056 (32.3)	1,169 (56.9)	887 (43.1)
60–69	2,179 (34.3)	878 (40.3)	1,301 (59.7)
≥70	652 (10.3)	216 (33.1)	436 (66.9)
BMI, kg/m^2^			
<24	3,245 (51.0)	2,063 (63.6)	1,182 (36.4)^**^
24 to <28	2,379 (37.4)	1,084 (45.6)	1,295 (54.4)
≥28	734 (11.5)	243 (33.1)	491 (66.9)
Educational level			
No	2,120 (33.3)	1,003 (47.3)	1,117 (52.7)^**^
Primary school	2,176 (34.2)	1,129 (51.9)	1,047 (48.1)
Middle school	1,483 (23.3)	917 (61.8)	566 (38.2)
High school and above	579 (9.1)	341 (58.9)	238 (41.1)
Current occupation			
Farmer or unemployment	4,612 (72.5)	2,317 (50.2)	2,295 (49.8)^**^
Blue-collar worker	646 (10.2)	401 (62.1)	245 (37.9)
Sales or service	390 (6.1)	248 (63.6)	142 (36.4)
Official job	642 (10.1)	386 (60.1)	256 (39.9)
Other	68 (1.1)	38 (55.9)	30 (44.1)
Current alcohol drinking	497 (7.8)	239 (48.1)	258 (51.9)^*^
Current smoking	1,116 (17.6)	610 (54.7)	506 (45.3)
Central obesity	2,204 (34.7)	846 (38.4)	1,358 (61.6)^**^
Physical activity			
Low	2,084 (32.8)	1,103 (52.9)	981 (47.1)^*^
Moderate	2,170 (34.1)	1,117 (51.5)	1,053 (48.5)
High	2,104 (33.1)	1,170 (55.6)	934 (44.4)
FLD			
No	4,106 (64.6)	2,504 (61.0)	1,602 (39.0)^**^
Mild	1,601 (25.2)	689 (43.0)	912 (57.0)
Moderate/severe	651 (10.2)	197 (30.3)	454 (69.7)
TC, mmol/L			
≤5.2	2,136 (33.6)	1,293 (60.5)	843 (39.5)^**^
>5.2	4,222 (66.4)	2,097 (49.7)	2,125 (50.3)
TG, mmol/L			
≤2.3	5,813 (91.4)	3,191 (54.9)	2,622 (45.1)^**^
>2.3	545 (8.6)	199 (36.5)	346 (63.5)
HDL-c, mmol/L			
≤2.0	5,552 (87.3)	2,921 (52.6)	2,631 (47.4)^*^
>2.0	806 (12.7)	469 (58.2)	337 (41.8)
LDL-c, mmol/L			
≤3.4	3,951 (62.1)	2,225 (56.3)	1,726 (43.7)^**^
>3.4	2,407 (37.9)	1,165 (48.4)	1,242 (51.6)

All data are shown as number (percentage).

FLD, fatty liver disease; TC, total cholesterol; TG, triglyceride; HDL-c, high-density lipoprotein cholesterol; LDL-c, low-density lipoprotein cholesterol.

^*^P-value <0.05.

^**^P-value <0.001.

### Association of hypertension with body composition

**Table 2** shows the associations between body composition and hypertension. The hypertensive population had significantly higher levels of BMI, WHR, WHtR, BFR, TC, TG, LDL-c, FTI, VAI, LAP, CMI, SM, LTI, bone weight, FBG, and FINS, but lower HDL-c and BMR than the normotensive. Then, the associations between various body compositions and hypertension were analyzed by univariable and multivariable logistic regression models. After adjustment for the potential confounding factors, including age, sex, current alcohol drinking, current smoking, and physical activity, indicators of fat-related anthropometric measurements (BMI, WHR, WHtR, and BFR), lipid metabolism (TC, TG, LDL-c, FTI, VAI, LAP, and CMI), and glucose metabolism (FBG and FINS) were positively associated with hypertension risk, while BMR was inversely associated. No significant association was observed for HDL-c, SM, LTI, and bone weight.

**Table 2:** The association between physical examination indicators, biochemical markers, and hypertension.

	Hypertension		Model 1 OR (95% CI)	Model 2 OR (95% CI)	Model 3 OR (95% CI)
	No (*n* = 3,390)	Yes (*n* = 2,968)			
BMI, kg/m^2^	23.42 ± 2.98	24.98 ± 3.31	1.68 (1.59–1.77)	1.71 (1.62–1.81)	1.70 (1.60–1.80)
<24	2,063 (60.9)	1,182 (39.8)			
<28	1,084 (32.0)	1,295 (43.6)	2.09 (1.87–2.32)	2.12 (1.89–2.37)	2.09 (1.87–2.35)
≥28	243 (7.2)	491 (16.5)	3.53 (2.98–4.18)	3.75 (3.13–4.48)	3.70 (3.10–4.43)
WHR	0.86 ± 0.07	0.90 ± 0.06	1.77 (1.68–1.87)	1.57 (1.48–1.67)	1.31 (1.22–1.40)
WHtR	0.50 ± 0.05	0.54 ± 0.06	1.97 (1.86–2.08)	1.78 (1.68–1.89)	1.56 (1.43–1.70)
BFR, %	27.44 ± 8.41	30.01 ± 8.82	1.35 (1.29–1.42)	2.18 (2.01–2.36)	1.80 (1.60–2.03)
TC, mmol/L	5.60 ± 1.06	5.84 ± 1.14	1.25 (1.19–1.31)	1.15 (1.09–1.22)	1.16 (1.09–1.22)
TG, mmol/L	1.20 ± 0.74	1.48 ± 0.90	1.49 (1.40–1.58)	1.47 (1.38–1.56)	1.32 (1.24–1.40)
HDL-c, mmol/L	1.62 ± 0.34	1.57 ± 0.36	0.87 (0.82–0.91)	0.84 (0.80–0.89)	0.96 (0.91–1.02)
LDL-c, mmol/L	3.17 ± 0.74	3.30 ± 0.77	1.19 (1.14–1.26)	1.09 (1.04–1.15)	1.07 (1.02–1.13)
FTI	6.58 ± 2.62	7.68 ± 3.03	1.49 (1.41–1.57)	1.95 (1.82–2.09)	1.80 (1.59–2.04)
VAI	1.34 ± 1.14	1.71 ± 1.41	1.41 (1.33–1.49)	1.41 (1.33–1.50)	1.25 (1.18–1.33)
LAP	26.06 ± 23.10	38.92 ± 30.19	1.76 (1.65–1.88)	1.69 (1.59–1.80)	1.42 (1.32–1.52)
CMI	0.42 ± 0.38	0.57 ± 0.47	1.50 (1.41–1.59)	1.47 (1.38–1.56)	1.26 (1.19–1.34)
BMR, %	52.90 ± 6.04	51.51 ± 6.01	0.79 (0.75–0.83)	0.56 (0.53–0.61)	0.70 (0.65–0.76)
SM, kg	41.00 ± 7.24	41.86 ± 7.95	1.12 (1.07–1.18)	1.49 (1.35–1.64)	0.99 (0.88–1.10)
LTI	15.90 ± 1.70	16.35 ± 1.84	1.29 (1.23–1.36)	1.61 (1.48–1.75)	0.99 (0.89–1.11)
Bone weight, kg	2.42 ± 0.37	2.44 ± 0.39	1.07 (1.02–1.13)	1.27 (1.19–1.36)	0.93 (0.86–1.01)
FBG, mmol/L	5.09 ± 1.32	5.60 ± 1.75	1.48 (1.39–1.58)	1.31 (1.23–1.39)	1.24 (1.17–1.32)
FINS, μU/ml	7.45 ± 4.83	9.11 ± 5.64	1.43 (1.35–1.52)	1.64 (1.54–1.75)	1.37 (1.28–1.47)
FLD					
No	2,504 (73.9)	1,602 (54.0)			
Mild	689 (20.3)	912 (30.7)	2.07 (1.84–2.33)	2.08 (1.84–2.35)	1.59 (1.39–1.81)
Moderate/severe	197 (5.8)	454 (15.3)	3.60 (3.01–4.31)	3.73 (3.10–4.50)	2.37 (1.93–2.92)

Continuous variables are described as mean ± SD, and categorical variables are shown as number (percentage). For continuous variables, the unit for the OR estimate is SD (calculated from the whole population).

Model 1, univariable model. Model 2 adjusted for sex and age (<40 years, 40–49 years, 50–59 years, 60–69 years, ≥70 years). Model 3 (full adjustment) for BMI further adjusted for current alcohol drinking (yes, no), current smoking (yes, no), and physical activity (low, moderate, high), in addition to those included in model 2. Model 3 for the other indicators further adjusted for BMI (<24.0 kg/m^2^, 24.0–28.0 kg/m^2^, ≥28.0 kg/m^2^), current alcohol drinking (yes, no), current smoking (yes, no), and physical activity (low, moderate, high), in addition to those included in model 2.

BMI, body mass index; WHR, waist-to-hip ratio; WHtR, waist-to-height ratio; BFR, body fat rate; TC, total cholesterol; TG, triglyceride; HDL-c, high-density lipoprotein cholesterol; LDL-c, low-density lipoprotein cholesterol; FTI, fat tissue index; VAI, visceral adiposity index; LAP, lipid accumulation product; CMI, cardiometabolic index; BMR, body moisture rate; SM, skeletal muscle; LTI, lean tissue index; FBG, fasting blood glucose; FINS, fasting insulin; FLD, fatty liver disease.

### Association of hypertension with body composition by the phenotypes of FLD

In the multivariable logistic regression analysis, the ORs (95% CI) of the mild FLD and moderate/severe FLD groups with hypertension were 1.59 (1.39–1.81) and 2.37 (1.93–2.92), when compared with the non-FLD group, after adjusting age, sex, BMI, current alcohol drinking, current smoking, and physical activity (**Table 2**).

Then, we stratified all participants into non-FLD, mild FLD, and moderate/severe FLD populations and analyzed the association between body composition and hypertension in multivariable adjusted logistic regression models in each stratum (**Table 3**). In the non-FLD population, the ORs of anthropometric indicators (BMI, WHR, WHtR, and BFR) with hypertension were 1.56 (1.44–1.70), 1.21 (1.11–1.32), 1.45 (1.30–1.61), and 1.57 (1.36–1.81), respectively. The increase of lipid metabolism-related indicators (TC, TG, LDL-c, FTI, VAI, LAP, and CMI) was related to higher risks of hypertension with ORs ranging from 1.09 to 1.68, while BMR was associated with a decreased risk of hypertension (OR: 0.77, 95% CI: 0.70–0.85). For indicators related to glucose metabolism, both FBG and FINS levels were significantly and positively associated with the risk of hypertension, with ORs of 1.21 (1.10–1.33) and 1.30 (1.17–1.45), respectively.

**Table 3:** Association between physical examination indicators, biochemical markers, and hypertension stratified by FLD grade.

	Model 3			*P*_For multiplicative interaction_
	Non-FLD	Mild FLD	Moderate/severe FLD	
BMI, kg/m^2^	1.56 (1.44–1.70)	1.45 (1.27–1.66)	1.10 (0.92–1.33)	0.003
WHR	1.21 (1.11–1.32)	1.22 (1.06–1.42)	1.37 (1.07–1.75)	0.815
WHtR	1.45 (1.30–1.61)	1.43 (1.18–1.73)	1.45 (1.07–1.95)	0.338
BFR, %	1.57 (1.36–1.81)	2.08 (1.56–2.75)	1.20 (0.77–1.86)	0.622
TC, mmol/L	1.18 (1.10–1.26)	1.08 (0.97–1.21)	0.98 (0.82–1.17)	0.183
TG, mmol/L	1.41 (1.28–1.56)	1.15 (1.05–1.27)	1.05 (0.92–1.19)	0.001
HDL-c, mmol/L	1.01 (0.94–1.08)	1.02 (0.90–1.16)	1.16 (0.92–1.46)	0.236
LDL-c, mmol/L	1.09 (1.01–1.16)	1.00 (0.90–1.12)	0.91 (0.76–1.09)	0.179
FTI	1.68 (1.43–1.97)	1.79 (1.37–2.34)	1.18 (0.86–1.62)	0.038
VAI	1.29 (1.17–1.43)	1.12 (1.02–1.23)	1.03 (0.91–1.16)	0.015
LAP	1.63 (1.43–1.86)	1.19 (1.06–1.32)	1.13 (0.98–1.29)	<0.001
CMI	1.33 (1.19–1.49)	1.11 (1.01–1.23)	1.04 (0.92–1.18)	0.004
BMR, %	0.77 (0.70–0.85)	0.67 (0.56–0.79)	0.87 (0.66–1.15)	0.925
SM, kg	1.04 (0.90–1.20)	0.69 (0.55–0.87)	1.13 (0.77–1.64)	0.429
LTI	1.05 (0.92–1.20)	0.75 (0.59–0.95)	1.10 (0.73–1.64)	0.166
Bone weight, kg	0.96 (0.87–1.07)	0.75 (0.64–0.89)	1.03 (0.78–1.35)	0.346
FBG, mmol/L	1.21 (1.10–1.33)	1.15 (1.05–1.26)	1.11 (0.95–1.29)	0.661
FINS, μU/ml	1.30 (1.17–1.45)	1.25 (1.11–1.40)	1.18 (1.01–1.39)	0.320

The unit for the OR estimate in model 3 is SD (calculated from the whole population). Model 3 for BMI adjusted for sex (male, female), age (<40 years, 40–49 years, 50–59 years, 60–69 years, ≥70 years), current alcohol drinking (yes, no), current smoking (yes, no), and physical activity (low, moderate, high). Model 3 for the other indicators further adjusted for BMI (<24.0 kg/m^2^, 24.0 to <28.0 kg/m^2^, ≥28.0 kg/m^2^). BMI, body mass index; WHR, waist-to-hip ratio; WHtR, waist-to-height ratio; BFR, body fat rate; TC, total cholesterol; TG, triglyceride; HDL-c, high-density cholesterol; LDL-c, low-density cholesterol; FTI, fat tissue index; VAI, visceral adiposity index; LAP, lipid accumulation product; CMI, cardiometabolic index; BMR, body moisture rate; SM, skeletal muscle; LTI, lean tissue index; FBG, fasting blood glucose; FINS, fasting insulin.

In the mild FLD population, the significant ORs of WHR and WHtR for hypertension were similar to those of the non-FLD population, whereas the ORs of BMI, BFR, TG, FTI, VAI, LAP, CMI, FBG, and FINS were also significant, but the strengths of associations showed a certain decrease in seven of the nine indicators. In contrast to the non-FLD, the OR of TC was non-significant, while the ORs of BMR, SM, LTI, and bone weight were all statistically, significantly lower than 1.00. Only the ORs of WHR, WHtR, and FINS were still significant and similar to those in non-FLD and mild FLD in the moderate/severe FLD population. The ORs of BMI and all the other indicators in the body composition profile lost statistical significance (95% CIs included 1.00) (**Table 3**).

### The interaction between sex, age, FLD, and body composition to hypertension risk

To investigate the combined effects of physical examination indicators, biochemical markers, and sex on hypertension risk, we applied multiplicative interaction analysis, and the results showed statistically significant multiplicative interactions between sex and BMI, BFR, SM, or bone weight (**Table S2**). The results of the interaction analysis between body composition and age to hypertension risk showed that WHR, BFR, TC, LDL-c, FTI, VAI, BMR, SM, LTI, and FINS all interacted significantly with age (**Table S3**).

Moreover, **Table 3** shows that the antagonistic multiplicative interactions between FLD and BMI, TG, FTI, VAI, LAP, or CMI were statistically significant.

### The trends in various body composition and hypertension risk in different FLD populations

**Figure 2:**
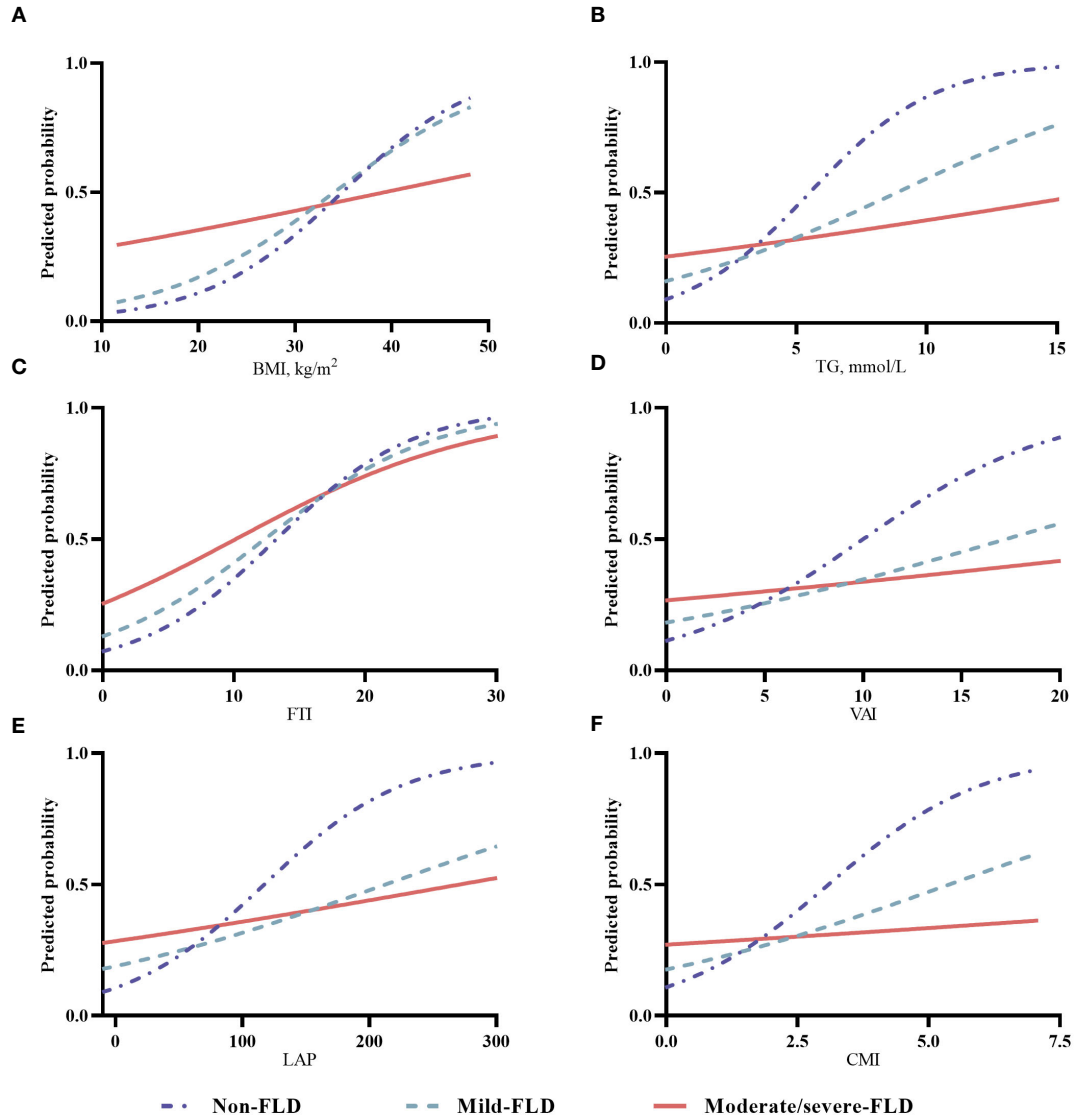
The trends in various body composition and hypertension risk in different FLD populations. **(A)** for BMI, body mass index; **(B)** for TG, triglyceride; **(C)** for FTI, fat tissue index; **(D)** for VAI, visceral adiposity index; **(E)** for LAP, lipid accumulation product; **(F)** for CMI, cardiometabolic index; (Notes: For BMI, the predicted probability of hypertension was adjusted for sex (male, female), age (<40, 40-49, 50-59, 60-69, ≥70 years), current alcohol drinking (yes, no), current smoking (yes, no), and physical activity (low, moderate, high). For other indicators, the predicted probability of hypertension was further adjusted for BMI (<24.0 kg/m2, 24.0-<28.0 kg/m2, ≥28.0 kg/m2).

To assess the adjusted trends in body composition and hypertension risk in different FLD populations, we calculated the predicted risks based on the multivariable adjusted logistic regression models. For the indicators significantly interacting with FLD, the associations between hypertension risk and various body composition indicators are plotted in **Figure 2**, and the trends for the other indicators are shown in **Figure S1**. For BMI, the predicted probability of hypertension increased sharply with greater BMI in the non-FLD population, increased less sharply in the mild FLD, but increased slightly in the moderate/severe FLD (**Figure 2A**). The trends for TG, FTI, VAI, LAP, and CMI (**Figures 2B–F**) varied among the different FLD populations. When these indicators were at low levels, participants with more severe FLD had a higher risk of hypertension. The risk of hypertension increased with these indicators, while the risk increased faster in the non-FLD population than in those with mild and moderate/severe FLD, and eventually, the risk of hypertension in the non-FLD population would even exceed that of those with moderate/severe FLD (**Figures 2B–F**). The trends for WHR, WHtR, and BFR in the non-FLD, mild FLD, and moderate/severe FLD populations were similar. They were positively associated with the predicted probability of hypertension, and the strengths of their associations were similar across the three populations (**Figures S1A–C**). The trend of hypertension risk with HDL-c varied in different populations. At the same HDL-c level, the risk of hypertension was consistently lower in the non-FLD group than in the mild FLD group. The risk was increasing in the moderate/severe FLD group, but there was little change or even a slight decrease in the risk in the mild FLD and non-FLD groups (**Figure S1E**). The predicted probability of hypertension decreased with higher BMR, SM, LTI, and bone weight (**Figures S1G–J**). The decrease was steepest in the non-FLD group, followed by the mild and moderate/severe FLD groups. The trends in hypertension risk for FBG and FINS varied across FLD populations, with a bit sharper increase in hypertension risk in the non-FLD population (**Figures S1K, L**).

### Association of hypertension with FLD stratified by body composition indicators

Stratified analyses were performed to determine the effect of each body composition indicator on the relationship between FLD and hypertension. The ORs of moderate/severe FLD for hypertension in the lowest tertile of BMI, WHR, WHtR, LAP, CMI, and LTI were all higher than the ORs in their middle and highest tertiles. Moreover, the ORs of moderate/severe FLD for hypertension were higher in the highest tertile of BFR, FTI, FBG, and FINS than in their middle and lowest tertiles (**Table S1**).

### The mediation effect of FLD in the association between body composition and hypertension

We performed a mediation analysis to evaluate the indirect effect of FLD in the association of each indicator of body composition with hypertension risk (**Table 4**). The indirect effect of FLD accounted for a non-negligible proportion (17.26%–38.90%) of the associations between the risk of hypertension and BMI, WHR, WHtR, BFR, TC, TG, LDL-c, FTI, VAI, LAP, CMI, BMR, FBG, and FINS.

**Table 4:** Mediation analysis with FLD as a potential mediator between physical examination indicators, biochemical markers, and hypertension risk.

Exposure variables	Hypertension, odds ratio (95% CI)			
	Natural direct effect	Natural indirect effect	Marginal total effect	Proportion, %^a^
BMI, kg/m^2^	1.48 (1.31–1.67)	1.17 (1.10–1.26)	1.73 (1.52–1.98)	29.01
WHR	1.24 (1.16–1.33)	1.08 (1.05–1.12)	1.34 (1.25–1.44)	26.28
WHtR	1.47 (1.34–1.60)	1.10 (1.05–1.15)	1.62 (1.47–1.77)	20.06
BFR, %	1.66 (1.47–1.87)	1.15 (1.08–1.22)	1.90 (1.67–2.17)	21.55
TC, mmol/L	1.13 (1.07–1.20)	1.03 (1.01–1.05)	1.16 (1.10–1.23)	17.26
TG, mmol/L	1.25 (1.17–1.33)	1.09 (1.05–1.13)	1.36 (1.27–1.45)	27.84
LDL-c, mmol/L	1.05 (0.99–1.11)	1.03 (1.01–1.05)	1.08 (1.02–1.15)	38.90
FTI	1.65 (1.45–1.87)	1.16 (1.08–1.24)	1.91 (1.67–2.18)	22.64
VAI	1.18 (1.11–1.26)	1.10 (1.06–1.14)	1.30 (1.21–1.39)	36.27
LAP	1.33 (1.23–1.43)	1.11 (1.06–1.17)	1.48 (1.37–1.60)	27.59
CMI	1.19 (1.11–1.27)	1.11 (1.06–1.15)	1.31 (1.23–1.41)	36.89
BMR, %	0.74 (0.68–0.80)	0.94 (0.90–0.97)	0.69 (0.63–0.75)	17.94
FBG, mmol/L	1.19 (1.12–1.26)	1.06 (1.03–1.09)	1.26 (1.18–1.34)	25.30
FINS, μU/ml	1.29 (1.20–1.38)	1.10 (1.05–1.14)	1.41 (1.31–1.52)	26.90

The unit for the OR estimate is SD (calculated from the whole population). For BMI, adjusted for sex (male, female), age (<40 years, 40–49 years, 50–59 years, 60–69 years, ≥70 years), current alcohol drinking (yes, no), current smoking (yes, no), and physical activity (low, moderate, high). The model for the other indicators further adjusted for BMI (<24.0 kg/m^2^, 24.0 to <28.0 kg/m^2^, ≥28.0 kg/m^2^). BMI, body mass index; WHR, waist-to-hip ratio; WHtR, waist-to-height ratio; BFR, body fat rate; TC, total cholesterol; TG, triglyceride; LDL-c, low-density cholesterol; FTI, fat tissue index; VAI, visceral adiposity index; LAP, lipid accumulation product; CMI, cardiometabolic index; BMR, body moisture rate; FBG, fasting blood glucose; FINS, fasting insulin.

a Proportion mediated was calculated as log(natural indirect relationship)/log(total relationship).

### Sensitivity analysis

A total of 31 individuals in the population included in this study underwent cholecystectomy. The results in **Table S2** indicate that there was no significant association between a history of cholecystectomy and FLD or hypertension. In addition, to minimize the influence of cholecystectomy history on the results, we performed a sensitivity analysis after excluding patients who underwent cholecystectomy. The results of the sensitivity analysis were highly consistent with those reported above (data not shown).

## Discussion

In the current study, we found that the body composition profile of the hypertensive population was different from that of the normotensive population. Among them, WHR, WHtR, BFR, FTI, VAI, LAP, CMI, FBG, and FINS were positively associated with hypertension risk, while BMR was inversely associated with the risk. The strength of the associations between body composition indicators and hypertension gradually decreased with the presence and severity of FLD, which was an independent risk factor for hypertension. FLD was an important mediator in the association between body composition profile and hypertension.

In general, BFR and WHR were the most commonly used indicators when assessing body composition and fat distribution (1). A cohort study indicated that the increase of fat mass, WC, and WHR predicted a higher hypertension risk, while maintenance of fat mass showed a lower risk. Moreover, hypertensive patients at baseline whose BP decreased after 10 years of follow-up showed a profound decrease in fat mass, even an increase of relative fat-free mass (10). Recently, SM and fat-free mass have been proposed. Their inverse association with hypertension has been reported (11), and their loss could partly explain the aging-associated risk of cardiovascular diseases and mortality (29). Additionally, the underlying causal associations between glucose metabolism and the risk of hypertension have been uncovered by Mendelian randomization studies (30). However, previous studies often focused on a few indicators of body composition, and a more comprehensive examination is needed. In our current study, a full description of body composition was given, including basic anthropometric measurements, BFR, lipid metabolism-related indicators, BMR, SM, and glucose metabolism indicators. The hypertensive population had higher BMI, WHR, WHtR, BFR, lipid metabolism, and glucose metabolism but lower BMR than the normotensive, showing a significantly different body composition.

Furthermore, inflammation, insulin resistance, and renin–angiotensin system–sympathetic nervous system activation were all critical pathophysiological mechanisms in the association between obesity and hypertension (31). Similarly, they also exist in the risk of FLD for hypertension (32). It has been reported that approximately 50% of hypertensive patients had FLD, and FLD patients had a significantly higher prevalence of hypertension (33–35). Similarly, the strong association between the presence and severity of FLD with increased hypertension risk was also significant in the current study. However, how FLD acts in the association between body composition and hypertension has not been examined. Therefore, we stratified all participants according to FLD status and investigated the association between body composition and hypertension. In the non-FLD population, various body composition indicators were all strongly associated with hypertension risk. In contrast, SM, LTI, and bone weight all showed an inverse association with hypertension in the mild FLD population. Previous studies judged them as favorable body composition (2, 7), and they may contribute to lower all-cause mortality and better prognosis of CVDs (2, 7, 36, 37). Notably, too, among moderate/severe FLD patients, only WHR, WHtR, and FINS were still significantly associated with hypertension, while neither lipid metabolism, glucose, nor favorable body composition was significantly associated.

To further confirm the results, the predicted curves were plotted. For WHR and WHtR, the predicted probability of hypertension increased almost linearly with their levels, and the strengths of association in the non-FLD, mild FLD, and moderate/severe FLD groups were similar. Indicators on lipid metabolism and glucose metabolism were all positively associated with hypertension risk, across the non-FLD, mild FLD, and moderate/severe FLD groups, while BMR, SM, and bone weight were inversely associated. The changing trends of the association between these indicators and hypertension were different among the three groups. On the whole, the trend changed mostly in the non-FLD group, followed by the mild FLD and moderate/severe FLD groups. Synthesizing all the results from the logistic regression analyses, we conceived that the association between whole body composition profile and hypertension varied largely with the phenotypes of FLD. We inferred that the underlying mechanisms of body composition on hypertension risk may be distinct in the presence or different severity of FLD, given that inflammation, insulin resistance, and even the whole body metabolism change when FLD occurs (38), and these should be explored further. Moreover, abdominal fat deposition, reflected by WHR and WHtR, was always important for hypertension, whether FLD existed or not.

Aside from the main results, we found that FLD was independently associated with hypertension, regardless of obesity and body composition. In the stratified analysis across all indicators on body composition profile, the moderate/severe FLD population had the highest prevalence of hypertension, followed by the mild FLD, when compared with the non-FLD. This was supported by the result adjusting the confounding effect of BMI in previous studies (33, 39). Interestingly, the ORs of moderate/severe FLD for hypertension were the highest in the lowest level of BMI, WHR, WHtR, and indicators on lipid metabolism, the second highest in their middle level, and the lowest in the highest level. All these suggested that the moderate/severe FLD population at the generally considered normal-weight levels may suffer a higher risk for hypertension than those with commonly defined obesity. This was similar to the result in a previous study that lean FLD patients showed a higher risk of hypertension than overweight/obese FLD patients (34). However, this should be further verified through large-scale cohort studies.

### Limitations

Our current study is subject to several limitations. First, this is a cross-sectional study that prohibits us from drawing causal associations between body composition, FLD, and hypertension. Second, all participants were recruited from local residents in southeast China, and the age and sex distribution of the current study were not possible to represent the natural population, which limited the generalizability of our results. Third, considering the practical feasibility, bioelectrical impedance technology using a convenient body composition meter was adopted in the measurement of the body composition profile. However, this method is limited by hydration status and is less accurate than whole-body scan using computed tomography or dual-energy X-ray absorptiometry equipment (40). Last, although multiple variables were adjusted in the regression models, the possibility of the existence of residual confounding and other unadjusted confounding factors cannot be excluded.

## Conclusions

The body composition profile of the hypertensive population was different from that of the normotensive. With the presence and severity of FLD, the association between body composition and hypertension was highly variable, and the observed association weakened gradually from the non-FLD to mild FLD populations and was non-significant in the moderate/severe FLD population. Moreover, FLD may be an important risk factor for hypertension, independent of BMI and body composition. However, FLD was associated with a higher excess risk in the normal-weight population than in the obese. FLD plays an important mediation role in obesity-associated hypertension. Further large-scale cohort and experimental studies are needed to validate the results and explore the potential mechanism.

## Data availability statement

The raw data supporting the conclusions of this article will be made available by the authors to any qualified researcher, without undue reservation.

## Ethics statement

The study has been approved by the Ethics Review Committee of Fujian Medical University (approval number, [2017-07] and [2020-58]), and the study protocol conforms to the ethical guidelines of the 1975 Declaration of Helsinki. Written informed consent was obtained from all participants.

## Author contributions

All authors were responsible for the study concept and design and contributed to the field investigation and data collection. WY and SD obtained the funding. WY, SD, and RF were responsible for data curation. XH did the statistical analysis. SD, XH, and YZ drafted the manuscript. All authors revised the manuscript for important intellectual content. All authors contributed to the article and approved the submitted version.

## Supplementary material

Supplementary Table 1 Association between FLD and hypertension stratified by physical examination indicators and biochemical markers.

Supplementary Table 2 Association between physical examination indicators, biochemical markers and hypertension stratified by sex.

Supplementary Table 3 Association between physical examination indicators, biochemical markers and hypertension stratified by age.

Supplementary Table 4 Association between history of cholecystectomy and hypertension as well as fatty liver disease (FLD).

Supplementary Figure 1 The trends in various body composition and hypertension risk in different FLD populations.
