# Supplementary material for: Associations between body composition profile and hypertension in different fatty liver phenotypes
Source: Front Endocrinol (Lausanne). 2023 Nov 28;14:1247110. doi: 10.3389/fendo.2023.1247110 (PMC10714003; doi:10.3389/fendo.2023.1247110)
Supplement: Supplementary Table 1 — Association between FLD and hypertension stratified by physical examination indicators and biochemical markers. [file Presentation_1.pdf]

frontiers

Supplementary Material

Associations between body composition profile and hypertension in different fatty liver phenotypes

Xiaoyin Huang[1†], Yuchen Zeng[2†], Mingyang Ma[1†], Liangguang Xiang[3], Qingdan Liu[1], Ling Xiao[1], Ruimei Feng[4], Wanxin Li[1], Xiaoling Zhang[2], Moufeng Lin[5], Zhijian Hu[1], Hongwei Zhao[6], Shanshan Du[1*], Weimin Ye[1, 7*]

[†] These authors contributed equally to this work and share first authorship.

[*] **Correspondence:** Weimin Ye and Shanshan Du, Fujian Medical University, Xuefunan Road 1, 350122, Fuzhou City, Fujian Province, China. Phone: +86 591 2286 2023; Fax: +86 591 2286 2510; Email: ywm@fjmu.edu.cn, dushanshan1007@163.com.

1 Supplementary Figures and Tables

1.1 Supplementary Tables

Table S1. Association between FLD and hypertension stratified by physical examination indicators and biochemical markers.

Stratification indicators	Model 1[a]		Model 2[b]		Model 3[c]	
	Mild	Moderate/severe	Mild	Moderate/severe	Mild	Moderate/severe
BMI, kg/m^2						
<24	1.87 (1.51-2.31)	5.07 (2.66-9.64)	1.83 (1.47-2.29)	5.14 (2.63-10.07)	1.82 (1.45-2.27)	5.07 (2.59-9.93)
<28	1.40 (1.18-1.67)	2.33 (1.78-3.04)	1.38 (1.15-1.66)	2.30 (1.74-3.03)	1.39 (1.16-1.67)	2.30 (1.74-3.04)
≥28	1.52 (1.01-2.27)	1.70 (1.13-2.55)	1.54 (1.00-2.37)	1.73 (1.12-2.67)	1.59 (1.03-2.46)	1.80 (1.16-2.79)
WHR						
T1	1.56 (1.19-2.05)	3.35 (1.86-6.02)	1.65 (1.24-2.21)	3.42 (1.84-6.35)	1.28 (0.94-1.75)	2.26 (1.17-4.37)
T2	1.63 (1.35-1.98)	2.77 (1.97-3.89)	1.77 (1.44-2.17)	3.17 (2.22-4.53)	1.49 (1.20-1.85)	2.44 (1.68-3.56)
T3	1.59 (1.30-1.94)	2.22 (1.73-2.83)	1.78 (1.45-2.19)	2.66 (2.06-3.45)	1.54 (1.24-1.91)	1.98 (1.48-2.63)
WHtR						
T1	1.13 (0.81-1.59)	4.82 (1.91-12.14)	1.24 (0.87-1.77)	7.54 (2.85-19.99)	1.16 (0.81-1.68)	7.05 (2.65-18.77)
T2	1.23 (1.02-1.49)	1.47 (1.01-2.14)	1.39 (1.14-1.70)	1.74 (1.17-2.57)	1.32 (1.08-1.63)	1.57 (1.05-2.34)
T3	1.67 (1.36-2.04)	2.21 (1.74-2.82)	1.80 (1.46-2.23)	2.64 (2.05-3.40)	1.73 (1.39-2.14)	2.40 (1.83-3.13)

BFR, %						
T1	1.82 (1.46-2.26)	2.27 (1.41-3.63)	1.85 (1.47-2.33)	2.63 (1.60-4.32)	1.47 (1.14-1.88)	1.78 (1.05-3.01)
T2	2.62 (2.09-3.27)	3.79 (2.71-5.29)	1.71 (1.32-2.21)	2.31 (1.53-3.50)	1.66 (1.27-2.15)	2.01 (1.31-3.10)
T3	1.60 (1.32-1.93)	3.09 (2.39-3.98)	1.49 (1.21-1.82)	2.66 (2.04-3.48)	1.42 (1.15-1.75)	2.31 (1.74-3.06)
TC, mmol/L						
≤5.2	2.12 (1.72-2.62)	4.28 (3.07-5.97)	1.99 (1.59-2.50)	4.23 (2.97-6.03)	1.43 (1.12-1.83)	2.50 (1.69-3.70)
>5.2	1.98 (1.72-2.29)	3.23 (2.61-3.99)	2.07 (1.79-2.40)	3.41 (2.74-4.25)	1.63 (1.39-1.91)	2.24 (1.76-2.87)
TG, mmol/L						
≤2.3	1.97 (1.74-2.23)	3.81 (3.10-4.68)	1.96 (1.72-2.24)	3.85 (3.09-4.78)	1.49 (1.29-1.71)	2.41 (1.90-3.05)
>2.3	1.83 (1.19-2.82)	1.89 (1.19-2.99)	1.88 (1.20-2.94)	2.04 (1.26-3.31)	1.65 (1.03-2.66)	1.61 (0.93-2.79)
HDL-c, mmol/L						
≤2.0	2.05 (1.81-2.32)	3.50 (2.91-4.20)	2.07 (1.82-2.35)	3.61 (2.98-4.38)	1.60 (1.39-1.84)	2.33 (1.88-2.88)
>2.0	2.23 (1.44-3.47)	7.66 (2.58-22.78)	2.14 (1.36-3.39)	8.55 (2.78-26.28)	1.55 (0.95-2.55)	5.12 (1.53-17.16)
LDL-c, mmol/L						
≤3.4	2.16 (1.86-2.52)	3.94 (3.10-5.03)	2.18 (1.85-2.56)	4.18 (3.23-5.41)	1.58 (1.32-1.89)	2.49 (1.88-3.32)
>3.4	1.84 (1.53-2.21)	3.00 (2.30-3.92)	1.90 (1.57-2.30)	3.09 (2.35-4.06)	1.57 (1.28-1.94)	2.19 (1.61-2.98)
FTI						
T1	1.68 (1.30-2.18)	2.16 (1.08-4.31)	1.62 (1.23-2.14)	2.53 (1.23-5.21)	1.37 (1.03-1.82)	1.76 (0.83-3.70)
T2	2.13 (1.74-2.60)	2.93 (2.10-4.10)	1.55 (1.22-1.96)	2.09 (1.42-3.08)	1.59 (1.25-2.01)	2.09 (1.41-3.11)
T3	1.69 (1.39-2.05)	3.03 (2.37-3.87)	1.55 (1.26-1.90)	2.64 (2.03-3.43)	1.47 (1.19-1.81)	2.30 (1.75-3.03)
VAI						
T1	2.27 (1.74-2.96)	3.93 (1.88-8.20)	2.23 (1.68-2.95)	4.49 (2.06-9.80)	1.49 (1.10-2.03)	2.21 (0.97-5.01)
T2	1.73 (1.41-2.11)	3.71 (2.54-5.40)	1.73 (1.40-2.15)	3.95 (2.66-5.88)	1.42 (1.13-1.78)	2.68 (1.76-4.09)
T3	1.51 (1.24-1.83)	2.28 (1.80-2.89)	1.54 (1.26-1.89)	2.35 (1.83-3.01)	1.36 (1.10-1.69)	1.85 (1.40-2.45)
LAP						
T1	1.47 (1.03-2.11)	7.31 (1.47-36.34)	1.55 (1.06-2.26)	9.86 (1.81-53.62)	1.28 (0.86-1.89)	8.18 (1.48-45.29)
T2	1.39 (1.15-1.69)	1.97 (1.30-2.98)	1.46 (1.19-1.79)	2.26 (1.47-3.50)	1.31 (1.06-1.62)	1.91 (1.23-2.98)
T3	1.30 (1.06-1.59)	1.92 (1.51-2.43)	1.40 (1.13-1.73)	2.12 (1.65-2.73)	1.31 (1.05-1.63)	1.82 (1.39-2.39)
CMI						
T1	2.27 (1.70-3.01)	5.49 (1.92-15.64)	2.28 (1.68-3.07)	6.75 (2.23-20.47)	1.64 (1.19-2.27)	3.84 (1.23-12.01)
T2	1.51 (1.24-1.84)	3.25 (2.22-4.77)	1.60 (1.30-1.97)	3.53 (2.37-5.26)	1.31 (1.05-1.64)	2.61 (1.73-3.95)
T3	1.42 (1.17-1.73)	2.04 (1.61-2.59)	1.48 (1.20-1.82)	2.19 (1.70-2.80)	1.33 (1.07-1.65)	1.73 (1.31-2.29)

BMR, %						
T1	1.80 (1.48-2.18)	3.25 (2.53-4.17)	1.52 (1.23-1.87)	2.54 (1.94-3.32)	1.35 (1.08-1.68)	2.04 (1.53-2.72)
T2	2.27 (1.85-2.79)	3.93 (2.78-5.56)	1.74 (1.38-2.19)	3.02 (2.06-4.44)	1.61 (1.27-2.05)	2.51 (1.67-3.78)
T3	1.90 (1.51-2.39)	2.35 (1.47-3.75)	1.91 (1.50-2.43)	2.62 (1.60-4.29)	1.41 (1.08-1.84)	1.44 (0.83-2.51)
SM, kg						
T1	2.50 (2.01-3.12)	4.46 (3.08-6.45)	2.18 (1.72-2.75)	3.59 (2.44-5.28)	1.74 (1.35-2.23)	2.47 (1.62-3.75)
T2	2.18 (1.78-2.67)	5.13 (3.81-6.90)	1.91 (1.53-2.37)	3.92 (2.86-5.37)	1.55 (1.23-1.96)	2.76 (1.96-3.87)
T3	1.70 (1.40-2.07)	2.24 (1.68-2.98)	1.95 (1.59-2.39)	3.05 (2.24-4.14)	1.38 (1.11-1.73)	1.62 (1.14-2.30)
LTI						
T1	2.22 (1.75-2.80)	5.26 (3.20-8.66)	1.86 (1.45-2.39)	4.11 (2.42-6.98)	1.61 (1.23-2.09)	3.23 (1.87-5.59)
T2	2.27 (1.86-2.77)	4.23 (3.19-5.59)	2.04 (1.65-2.53)	3.41 (2.53-4.59)	1.78 (1.42-2.22)	2.69 (1.96-3.69)
T3	1.54 (1.27-1.87)	2.21 (1.68-2.91)	1.78 (1.45-2.17)	2.86 (2.13-3.82)	1.31 (1.05-1.63)	1.62 (1.17-2.26)
Bone weight, kg						
T1	2.44 (1.87-3.17)	4.80 (2.99-7.71)	2.14 (1.62-2.83)	4.01 (2.46-6.56)	1.72 (1.28-2.32)	2.80 (1.65-4.73)
T2	2.25 (1.85-2.72)	4.94 (3.64-6.69)	2.06 (1.67-2.53)	3.93 (2.85-5.41)	1.70 (1.37-2.12)	2.83 (2.01-3.99)
T3	1.74 (1.45-2.09)	2.52 (1.95-3.26)	1.85 (1.53-2.24)	3.06 (2.33-4.02)	1.36 (1.10-1.67)	1.77 (1.30-2.42)
FBG, mmol/L						
T1	1.86 (1.49-2.33)	2.54 (1.73-3.74)	1.89 (1.49-2.40)	2.81 (1.87-4.23)	1.26 (0.97-1.64)	1.52 (0.98-2.37)
T2	1.76 (1.43-2.15)	2.91 (2.07-4.08)	1.76 (1.42-2.18)	3.04 (2.13-4.34)	1.40 (1.11-1.76)	1.87 (1.25-2.80)
T3	1.92 (1.58-2.34)	3.19 (2.44-4.17)	2.13 (1.73-2.62)	3.73 (2.83-4.93)	1.76 (1.42-2.20)	2.77 (2.04-3.77)
FINS, μU/ml						
T1	1.80 (1.38-2.34)	2.43 (1.06-5.58)	1.75 (1.33-2.31)	2.14 (0.91-5.05)	1.32 (0.98-1.76)	1.12 (0.46-2.74)
T2	1.60 (1.31-1.95)	2.13 (1.49-3.04)	1.38 (1.12-1.71)	1.70 (1.17-2.48)	1.21 (0.96-1.52)	1.34 (0.90-2.00)
T3	2.10 (1.73-2.56)	3.56 (2.79-4.54)	1.91 (1.54-2.35)	3.26 (2.52-4.21)	1.70 (1.37-2.12)	2.65 (2.00-3.51)

Abbreviation: WHR: waist-to-hip ratio; WHtR, waist-to-height ratio; BFR, body fat rate; TC, total cholesterol; TG, triglyceride; HDL-c, high-density lipoprotein cholesterol; LDL-c, low-density lipoprotein cholesterol; FTI, fat tissue index; VAI, visceral adiposity index; LAP, lipid accumulation product; CMI, cardiometabolic index; BMR, body moisture rate; SM, skeletal muscle; LTI, lean tissue index; FBG, fasting blood glucose; FINS, fasting insulin.

The reference was non-FLD group in the same level of stratification indicators

[a]Model 1, univariable model

[b]Model 2, adjusted for sex, age (<40, 40-49, 50-59, 60-69, ≥70 years)

[c]Model 3 (full adjustment), for BMI further adjusted for current alcohol drinking (yes, no), current smoking (yes, no), physical activity (low, moderate, high) in addition to those included in model 2. Model 3 for other indicators further adjusted for BMI (<24.0 kg/m^2, 24.0-28.0 kg/m^2, ≥28.0 kg/m2), current alcohol drinking (yes, no), current smoking (yes, no), physical activity (low, moderate, high) in addition to those included in model 2

Table S2 Association between physical examination indicators, biochemical markers and hypertension stratified by sex

	Model 3		*P* for multiplicative interaction
	Male	Female	
BMI, kg/m^2	1.79 (1.62-1.97)	1.61 (1.50-1.73)	0.012
WHR	1.25 (1.11-1.40)	1.29 (1.18-1.40)	0.943
WHtR	1.51 (1.30-1.75)	1.52 (1.36-1.70)	0.446
BFR, %	1.99 (1.65-2.40)	1.73 (1.48-2.02)	0.001
TC, mmol/L	1.11 (1.01-1.21)	1.16 (1.08-1.24)	0.284
TG, mmol/L	1.18 (1.08-1.30)	1.38 (1.27-1.49)	0.115
HDL-c, mmol/L	0.97 (0.87-1.07)	0.97 (0.90-1.04)	0.238
LDL-c, mmol/L	1.02 (0.93-1.12)	1.08 (1.00-1.15)	0.261
FTI	2.29 (1.83-2.87)	1.75 (1.50-2.03)	<0.001
VAI	1.17 (1.05-1.31)	1.26 (1.17-1.35)	0.998
LAP	1.27 (1.14-1.43)	1.46 (1.33-1.60)	0.526
CMI	1.14 (1.04-1.25)	1.32 (1.21-1.44)	0.165
BMR, %	0.71 (0.64-0.79)	0.71 (0.64-0.80)	0.067
SM, kg	1.04 (0.89-1.20)	0.79 (0.66-0.95)	<0.001
LTI	0.95 (0.81-1.10)	0.92 (0.77-1.10)	0.025
Bone weight, kg	1.06 (0.92-1.23)	0.88 (0.80-0.97)	<.0001
FBG, mmol/L	1.21 (1.10-1.33)	1.24 (1.15-1.34)	0.408
FINS, μU/ml	1.27 (1.13-1.42)	1.40 (1.29-1.53)	0.607

Note: The unit for OR estimate in Model 3 is SD (calculated from the whole population). Model 3 for BMI, adjusted for age (<40, 40-49, 50-59, 60-69, ≥70 years), current alcohol drinking (yes, no), current smoking (yes, no), and physical activity (low, moderate, high). Model 3 for other indicators further adjusted for BMI (<24.0 kg/m^2, 24.0-<28.0 kg/m^2, ≥28.0 kg/m^2). WHR, waist-to-hip ratio; WHtR, waist-to-height ratio; BFR, body fat rate; TC, total cholesterol; TG, triglyceride; HDL-c, high-density cholesterol; LDL-c, low-density cholesterol; FTI, fat tissue index; VAI, visceral adiposity index; LAP, lipid accumulation product; CMI, cardiometabolic index; BMR, body moisture rate; SM, skeletal muscle; LTI, lean tissue index; FBG, fasting blood glucose; FINS, fasting insulin.

Table S3 Association between physical examination indicators, biochemical markers and hypertension stratified by age

	Model 3		P for multiplicative interaction
	<60 years	60+ years	
BMI, kg/m2	1.69 (1.57-1.82)	1.71 (1.57-1.87)	0.707
WHR	1.51 (1.37-1.66)	1.23 (1.12-1.36)	0.002
WHtR	1.78 (1.58-2.01)	1.54 (1.36-1.74)	0.366
BFR, %	1.79 (1.51-2.11)	1.94 (1.65-2.29)	0.005
TC, mmol/L	1.31 (1.21-1.41)	1.11 (1.03-1.20)	0.013
TG, mmol/L	1.27 (1.18-1.37)	1.42 (1.28-1.58)	0.118
HDL-c, mmol/L	1.06 (0.98-1.14)	0.92 (0.85-1.01)	0.253
LDL-c, mmol/L	1.20 (1.11-1.29)	1.04 (0.96-1.12)	0.018
FTI	1.81 (1.53-2.14)	1.95 (1.63-2.34)	0.023
VAI	1.20 (1.12-1.29)	1.37 (1.22-1.53)	0.028
LAP	1.37 (1.26-1.49)	1.55 (1.38-1.76)	0.112
CMI	1.21 (1.12-1.30)	1.37 (1.22-1.53)	0.135
BMR, %	0.76 (0.68-0.85)	0.70 (0.63-0.78)	0.022
SM, kg	0.79 (0.68-0.92)	0.94 (0.81-1.10)	0.017
LTI	0.94 (0.80-1.09)	0.95 (0.82-1.10)	0.007
Bone weight, kg	0.82 (0.74-0.91)	0.92 (0.82-1.04)	0.188
FBG, mmol/L	1.29 (1.19-1.41)	1.25 (1.15-1.36)	0.661
FINS, μU/ml	1.23 (1.13-1.33)	1.46 (1.31-1.64)	0.007

Note: The unit for OR estimate in Model 3 is SD (calculated from the whole population). Model 3 for BMI, adjusted for sex (male, female), current alcohol drinking (yes, no), current smoking (yes, no), and physical activity (low, moderate, high). Model 3 for other indicators further adjusted for BMI (<24.0 kg/m^2, 24.0-<28.0 kg/m^2, ≥28.0 kg/m^2). WHR, waist-to-hip ratio; WHtR, waist-to-height ratio; BFR, body fat rate; TC, total cholesterol; TG, triglyceride; HDL-c, high-density cholesterol; LDL-c, low-density cholesterol; FTI, fat tissue index; VAI, visceral adiposity index; LAP, lipid accumulation product; CMI, cardiometabolic index; BMR, body moisture rate; SM, skeletal muscle; LTI, lean tissue index; FBG, fasting blood glucose; FINS, fasting insulin.

Table S4. Association between history of cholecystectomy and hypertension as well as fatty liver disease (FLD).

	Total	Cholecystectomy		*P*
		No (n=6327)	Yes (n=31)	
Hypertension				0.849
No	3390(53.3)	3374(53.3)	16(51.6)	
Yes	2968(46.7)	2953(46.7)	15(48.4)	
FLD				0.154
No	4106(64.6)	4091(64.7))	15(48.4)	
Mild	1601(25.2)	1589(25.1)	12(38.7)	
Moderate/severe	651(10.2)	647(10.2)	4(12.9)	

Abbreviation: FLD, fatty liver disease.

1.2 Supplementary Figures

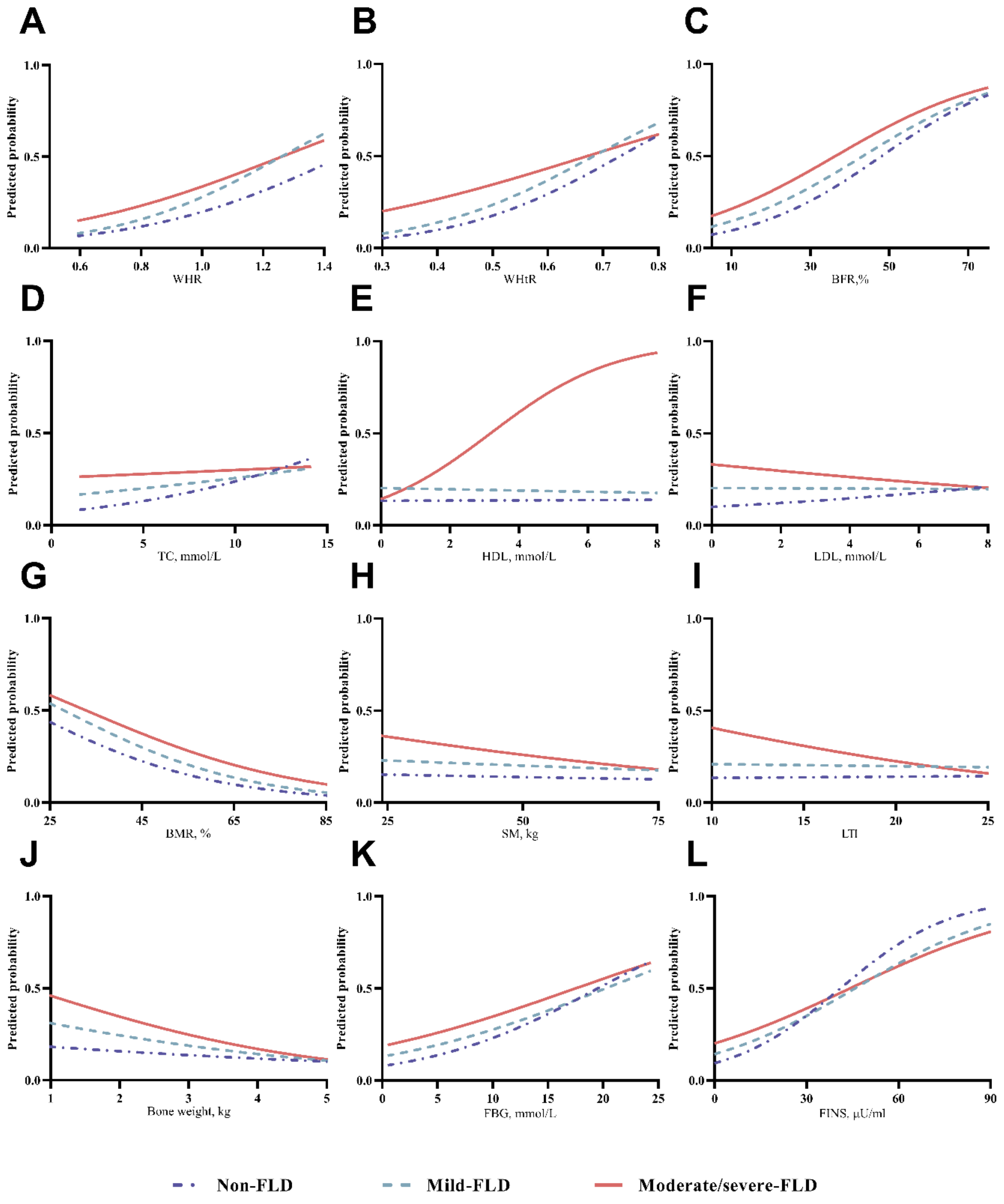

Figure S1. The trends in various body composition and hypertension risk in different FLD populations The predicted probability of hypertension was adjusted for sex (male, female), age (<40, 40-49, 50-59, 60-69, ≥70 years), BMI (<24.0 kg/m^2, 24.0-<28.0 kg/m^2, ≥28.0 kg/m^2), current alcohol drinking (yes, no), current smoking (yes, no), and physical activity (low, moderate, high)

Abbreviation: WHR, waist-to-hip ratio; WHtR, waist-to-height ratio; BFR, body fat rate; TC, total cholesterol; HDL-c, high-density lipoprotein cholesterol; LDL-c, low-density lipoprotein cholesterol; BMR, body moisture rate; SM, skeletal muscle; LTI, lean tissue index; FBG, fasting blood glucose; FINS, fasting insulin.
